# Low expression or hypermethylation of PLK2 might predict favorable prognosis for patients with glioblastoma multiforme

**DOI:** 10.7717/peerj.7974

**Published:** 2019-11-19

**Authors:** Xiangping Xia, Fang Cao, Xiaolu Yuan, Qiang Zhang, Wei Chen, Yunhu Yu, Hua Xiao, Chong Han, Shengtao Yao

**Affiliations:** 1 Department of Cerebrovascular Disease, The First Affiliated Hospital of Zunyi Medical University, Zunyi, Guizhou, China; 2 Department of Stroke Unit and Neurosurgery, The First People’s Hospital of Zunyi, Zunyi, Guizhou, China

**Keywords:** PLK2, Methylation, Prognosis, Glioblastoma multiforme, Overall survival

## Abstract

**Background:**

As the most aggressive brain tumor, patients with glioblastoma multiforme (GBM) have a poor prognosis. Our purpose was to explore prognostic value of Polo-like kinase 2 (PLK2) in GBM, a member of the PLKs family.

**Methods:**

The expression profile of PLK2 in GBM was obtained from The Cancer Genome Atlas database. The PLK2 expression in GBM was tested. Kaplan–Meier curves were generated to assess the association between PLK2 expression and overall survival (OS) in patients with GBM. Furthermore, to assess its prognostic significance in patients with primary GBM, we constructed univariate and multivariate Cox regression models. The association between PLK2 expression and its methylation was then performed. Differentially expressed genes correlated with PLK2 were identified by Pearson test and functional enrichment analysis was performed.

**Results:**

Overall survival results showed that low PLK2 expression had a favorable prognosis of patients with GBM (*P*-value = 0.0022). Furthermore, PLK2 (HR = 0.449, 95% CI [0.243–0.830], *P*-value = 0.011) was positively associated with OS by multivariate Cox regression analysis. In cluster 5, DNA methylated PLK2 had the lowest expression, which implied that PLK2 expression might be affected by its DNA methylation status in GBM. PLK2 in CpG island methylation phenotype (G-CIMP) had lower expression than non G-CIMP group (*P* = 0.0077). Regression analysis showed that PLK2 expression was negatively correlated with its DNA methylation (*P* = 0.0062, Pearson *r* = −0.3855). Among all differentially expressed genes of GBM, CYGB (*r* = 0.5551; *P* < 0.0001), ISLR2 (*r* = 0.5126; *P* < 0.0001), RPP25 (*r* = 0.5333; *P* < 0.0001) and SOX2 (*r* = −0.4838; *P* < 0.0001) were strongly correlated with PLK2. Functional enrichment analysis results showed that these genes were enriched several biological processes or pathways that were associated with GBM.

**Conclusion:**

Polo-like kinase 2 expression is regulated by DNA methylation in GBM, and its low expression or hypermethylation could be considered to predict a favorable prognosis for patients with GBM.

## Introduction

As the most aggressive brain tumor, glioblastoma multiforme (GBM) patients have a poor prognosis, with a median survival of about 14 months. GBM has the characteristics of obvious heterogeneity, rapid proliferation and extensive invasion (Stathias et al., 2018; Alifieris & Trafalis, 2015; Omuro & DeAngelis, 2013). According to gene expression studies from The Cancer Genome Atlas (TCGA), GBM are classified into four distinct molecular subtypes, including mesenchymal, classical, neural and proneural (Kloosterhof et al., 2013; Yin et al., 2018). The proneural subtype is more common in younger GBM patients and is associated with a better prognosis (Slotty et al., 2013). The classical and mesenchymal subtypes appear to be more sensitive to chemotherapy. Furthermore, the neural subtype has genetic phenotype most like normal brain (Ma et al., 2017). However, the genetic and/or epigenetic differences that lead to different tumor behaviors are still not fully understood. Even through the current standard treatment, the median survival of GBM patients is only 14.6 months (Han & Puri, 2018). Thus, there is an urgent need to elucidate the molecular mechanisms of GBM and to identify novel and effective therapeutic strategies for patients with GBM (Mao et al., 2017).

The protein encoded by PLK2 gene is a member of the polo-like kinase (PLK) of serine/threonine protein kinases (PLK1, PLK2, PLK3, PLK4 and PLK5) (Pezuk et al., 2013). High PLK2 rexpression has been confirmed in osteosarcomas (Shen et al., 2012), and down-regulated PLK2 could be associated with chemotherapy resistance in epithelial ovarian cancer (Syed et al., 2011). Furthermore, PLK2 is a direct target for transcriptional regulation by p53 (Feng et al., 2007). It has been identified that a new connection between the p53 tumor suppressor pathway and the oncogenic mTOR pathway through PLK2 in colorectal carcinomas (Matthew et al., 2018; Budanov & Karin, 2008). However, the prognostic value of PLK2 in GBM is still unclear and the underlying mechanisms of its dysregulation in GBM have been not fully understood. In our study, we hypothesized that PLK2 (logFC = −2.09880795 in RNA-seq data) was abnormally expressed in GBM.

DNA methylation markers have long been major candidates for cancer biomarker discovery (Issa, 2012). Epigenetic abnormalities play a crucial role in determining tumor phenotypic behavior by regulating gene expression and chromatin organization (Rodriguez-Paredes & Esteller, 2011). Early candidate gene approach studies have identified a number of DNA methylation changes in key genes that have the potential clinical value in GBM (Malzkorn et al., 2011). Whole genome methylation may have prognostic value in GBM. Tumors with relatively high DNA methylation at the CpG site have a more favorable prognosis than tumors with relatively lower methylation levels. Hypermethylation of oncogenes has been characterized as a favorable indicator for patients with GBM (Capper et al., 2018; Taylor, 2010). One study reported that PLK2 is hypermethylated in acute myeloid leukemia and myelodysplastic syndromes, and its hypermethylation is associated with a better prognosis (Benetatos et al., 2011). In our study, we explored whether PLK2 expression was in association with DNA methylation in GBM.

In the present study, we analyzed the prognostic value of PLK2 in GBM. Furthermore, we also examined its expression in different subtypes, DNA copy number alterations of GBM and explored its association with DNA methylation and CIMP.

## Materials and Methods

### Patients and datasets

The data of patients with GBM and the corresponding non-tumor normal controls were retrieved from TCGA-GBM project (http://cancergenome.nih.gov/), including DNA copy number alterations, DNA methylation, gene expression, and patient clinical information for GBM. This cohort included 602 biospecimens of primary GBM tumors. Among the 602 cases of primary tumors, 529 had PLK2 expression measured by RNA array (AffyU133a), and 154 had PLK2 expression measured by RNAseq (polyA+ Illumina HiSeq). A total of 152 of the 502 cases had intact clinical information, which were used for survival analysis. The clinicopathological parameters of the 152 patients in this cohort, including age, gender, Karnofsky Performance Score (KPS), radiation therapy, temozolomide chemotherapy and living status were downloaded from TCGA via UCSC Xena Browser (http://xena.ucsc.edu/). The UCSC Xena Browser is a bioinformatics tool to visualize functional genomics data from many sources, including TCGA database.

### The association between PLK2 expression and OS in patients with GBM

We analyzed the PLK2 expression on AffyU133a (529 GBM tumor tissues vs. 10 corresponding non-tumor tissues) and IlluminaHiSeq platform (154 GBM tumor vs. five corresponding non-tumor tissues), respectively. Firstly, the PLK2 mRNA expression was normalized by log2 (norm_count+1). Then, the patients in this cohort were divided into high expression and low expression groups based on the median PLK2 expression. Kaplan–Meier curves of overall survival (OS) were generated using GraphPad Prism 7.0 (GraphPad Software, Inc., San Diego, CA, USA). Log-rank test was used to evaluate the significant of the difference between the Kaplan–Meier curves.

The 276 GBM tumor samples and eight corresponding non-tumor samples from Gene Expression Omnibus (GEO; http://www.ncbi.nlm.nih.gov/geo/) dataset GSE16011 were used to validate the expression of PLK2 in GBM (Gravendeel et al., 2009). Moreover, the expression levels of other PLK family members (PLK1, PLK3 and PLK4) were analyzed in GSE16011 datasets.

### The expression levels of PLK family members across different subtypes and DNA copy number alterations of GBM

We evaluated the expression levels of PLK family members (PLK1, PLK2, PLK3 and PLK4) across four distinct molecular subtypes of GBM including proneural (*n* = 37), neural (*n* = 26), classical (*n* = 39) and mesenchymal (*n* = 49) subtypes. Furthermore, we analyzed the expression levels of PLK1, PLK2, PLK3 and PLK4 across different DNA copy number alterations, including shallow deletion (also known as hemizygosity; *n* = 30), diploid (*n* = 109) and gain (*n* = 8). The types of copy number variants have been defined as follows: amplification (copy number ≥ 4), gain (copy number > 2), shallow deletion (copy number 0).

### The associations between PLK2 expression and DNA methylation subtypes, DNA methylation and CpG methylation phenotypes in GBM

By data mining from TCGA-GBM using UCSC Xena Browser, the associations of between PLK2 expression and DNA methylation subtypes (cluster1–6), DNA methylation and CpG methylation phenotypes (G-CIMP and non-CIMP) in GBM were analyzed. The methylation related analysis was to give an overall map. Furthermore, we also assessed the associations between the expression levels of other PLK family members (PLK1, PLK3 and PLK4) and DNA methylation subtypes (cluster1–6), DNA methylation and CpG methylation phenotypes (G-CIMP and non-CIMP) in GBM.

### PLK2-related genes analysis

Using GEO dataset GSE16011, 3,089 differentially expressed genes (1,585 up-regulated and 1,504 down-regulated) of GBM with false discovery rate (FDR) < 0.05 and |log2FC| > 1 were identified. To determine PLK2-related genes in GBM, Pearson test was used to analyze correlations between PLK2 and differentially expressed genes. The Pearson correlation coefficients were then calculated. Volcano plots of PLK2-related genes were generated using ggplot2 package in R. The top 50 positively correlated genes and negatively correlated genes were shown using pheatmap package in R, respectively.

### Gene set enrichment analysis

To explore the biological processes and pathways enriched by genes associated with PLK2 in GBM, the Gene Ontology annotations and Kyoto Encyclopedia of Genes and Genomes (KEGG) pathways were performed using gene set enrichment analysis (GSEA) (http://software.broadinstitute.org/gsea/index.jsp) (Subramanian et al., 2005). The FDR < 0.05, |enrichment score | > 0.6 and gene size ≥100 were set as the cut-off criteria.

### Statistical analysis

All statistical analyses were performed using GraphPad Prism 7.0 (GraphPad Software, Inc.), SPSS 23.0 (IBM SPSS Statistics) and R language. The association between PLK2 expression and the clinicopathological parameters of 152 patients with primary GBM was evaluated by chi test using SPSS 23.0 (IBM SPSS Statistics, Chicago, IL, USA). Univariate and multivariate Cox regression models were used to assess the prognostic significance of PLK2 expression in GBM. Regression analyses was used to analyze the correlation between PLK2 expression and its DNA methylation. The comparisons between two groups were analyzed using the student’s *t*-test. For pairwise multiple comparisons, one-way ANOVA, followed by Dunnett’s multiple comparison test was performed. *P*-value < 0.05 was considered to be statistically significant.

## Results

### Lowly expressed PLK2 is associated with better OS of patients with GBM

**Figure 1:**
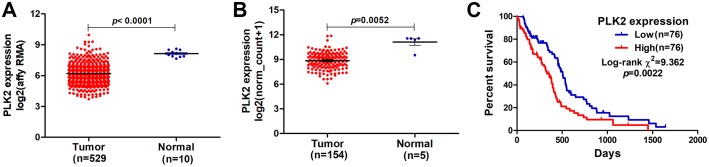
Lowly expressed PLK2 is associated with better OS of patients with GBM. (A) PLK2 expression was down-regulated in GBM tissues (*n* = 529) compared with normal non-tumor samples (*n* = 10) on AffyU133a platform (*P*-value < 0.001); (B) PLK2 expression was down-regulated in GBM tissues (*n* = 154) compared with normal non-tumor tissues (*n* = 5) on IlluminaHiSeq platform (*P*-value < 0.0052); (C) Survival analysis showed the association between the PLK2 expression and OS of 152 patients with GBM (*P*-value = 0.0022).

**Figure 2:**
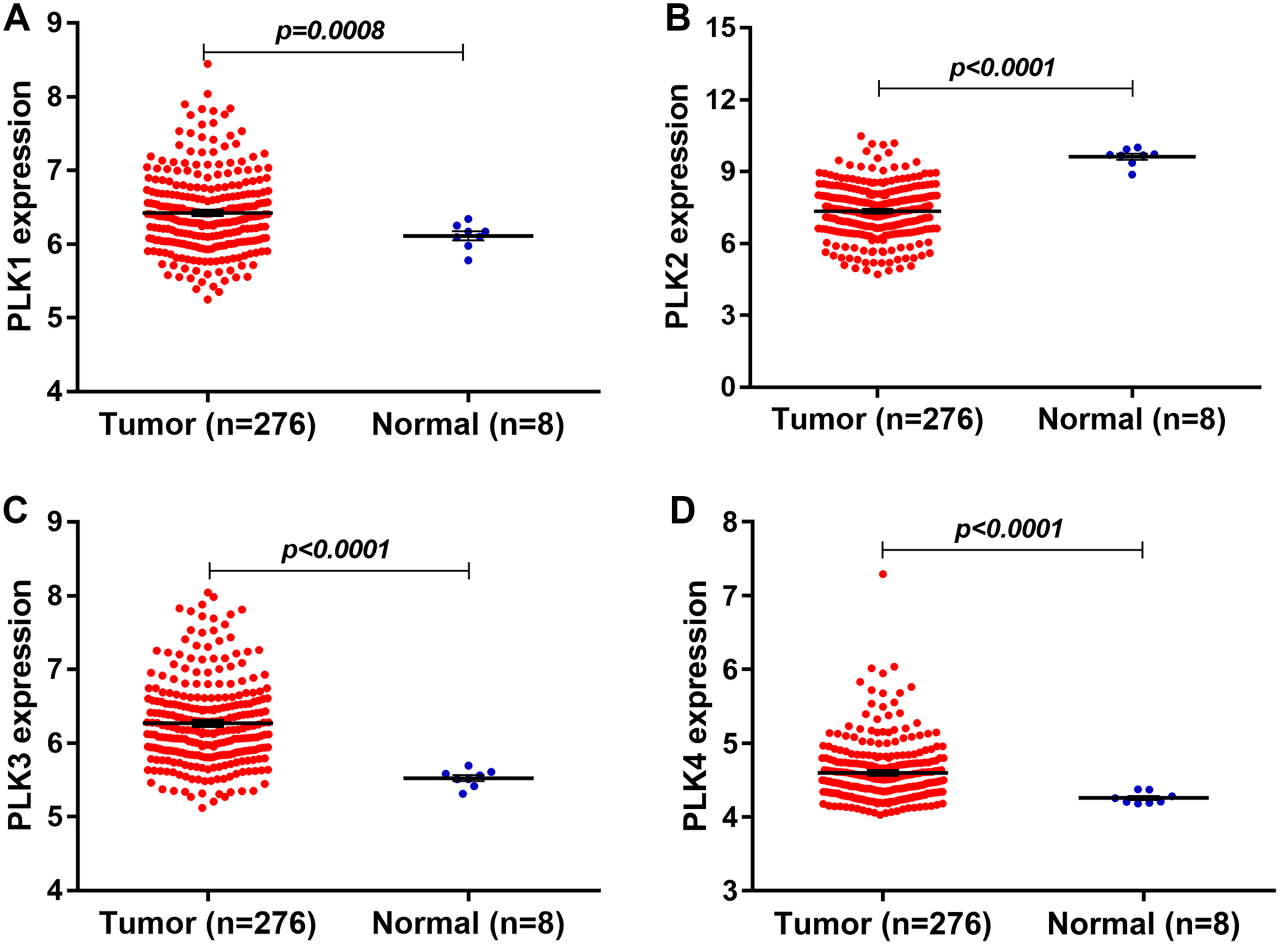
The expression levels of PLK family members in GBM using GSE16011 dataset. (A–D) PLK1 (*P* = 0.0008), PLK3 (*P* < 0.0001) and PLK4 (*P* < 0.0001) was all up-regulated in GBM tissues (*n* = 276) compared with the corresponding non-tumor tissues (*n* = 8); (B) The low PLK2 expression was validated in an independent GSE16011 dataset (*P* < 0.0001).

In our study, we found that PLK2 was significantly down-regulated in 529 GBM tissues compared to 10 normal non-tumor tissues (log_2_FC = −1.479; *P*-value < 0.0001; Fig. 1A) on AffyU133a platform. Similarly, PLK2 expression was significantly lower in 154 GBM tumor tissues than that in five corresponding non-tumor tissues on IlluminaHiSeq platform (*P* < 0.0052; Fig. 1B). Furthermore, survival analysis results indicated that patients with low PLK2 expression had longer OS time (*P* = 0.0022) in Fig. 1C. An independent dataset (GSE16011) was used to validate the expression of PLK2 in GBM. We found that PLK1 was up-regulated in GBM (logFC = 0.41167184; *P* = 0.0008; Fig. 2A). As expected, PLK2 was down-regulated in GBM (logFC = −2.09880795; *P* < 0.0001; Fig. 2B). Above results revealed that PLK2 was down-regulated in GBM and low PLK2 expression indicated a better prognosis of patients with GBM.

The expression levels of other PLK family members PLK3 (logFC = 0.92138804; *P* < 0.0001) and PLK4 (logFC = 0.40736722; *P* < 0.0001) was up-regulated in GBM (*n* = 276) compared with normal non-tumor tissues (*n* = 8) in Figs. 2C and 2D.

### PLK2 might be an independent prognostic factor for patients with GBM

A total of 152 GBM patients with complete follow-up information were included in this cohort (Table 1). These patients were divided into high and low PLK2 expression groups based on the median value of PLK2 expression. The associations between PLK2 expression and the clinicopathological parameters of GBM patients are shown in Table 1. The low PLK2 expression group had a higher KPS score (73.82 ± 14.075 vs. 77.00 ± 14.764), however, there was no statistically significant (*P*-value = 0.831 and 0.240, respectively). Furthermore, the low PLK2 group had more patients who accepted radiation therapy (54 vs. 59; *P*-value = 0.0490).

**Table 1:** Demographic and clinicopathological parameters of patients with primary GBM in TCGA-GBM.

Parameters		PLK2 expression		*P*-value
		High (*n* = 76)	Low (*n* = 76)	
Age (mean ± SD)		59.61 ± 12.827	60.08 ± 14.387	0.831
Gender	Female	24	30	0.309
	Male	52	46	
KPS score		73.82 ± 14.075	77.00 ± 14.764	0.240
Radiation therapy	True	54	59	0.049
	False	21	10	
	Null	7	7	
Temozolomide chemotherapy	True	49	49	0.465
	False	29	20	
	Null	1	7	
Living status	Living	16	21	0.345
	Dead	60	55	

**Note:**

KPS, Karnofsky performance score.

**Figure 3:**
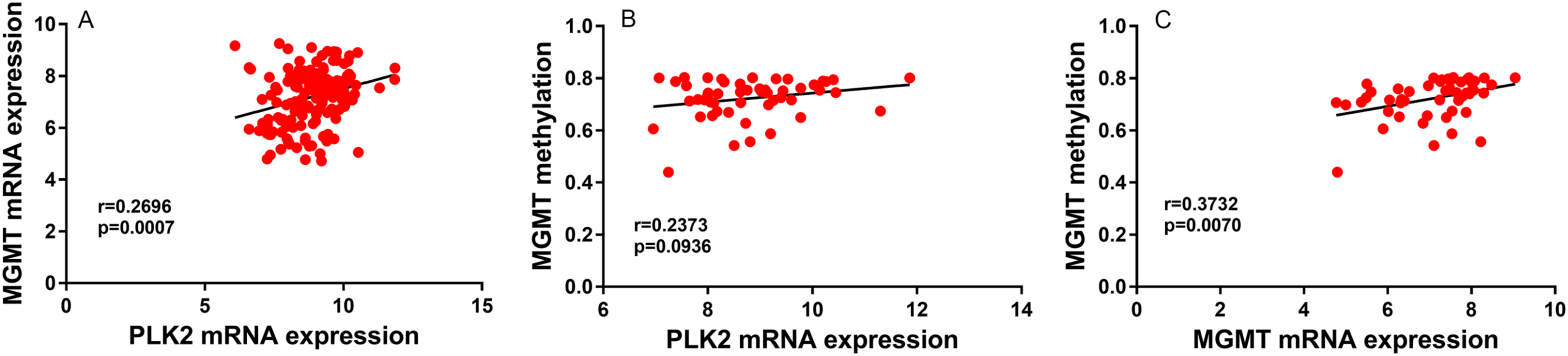
PLK2 expression may not be associated with MGMT methylation in GBM. (A) The correlation between PLK2 expression and MGMT expression. (B) The correlation between PLK2 expression and MGMT methylation. (C) The correlation between MGMT expression and its methylation.

In univariate analysis, age and high PLK2 expression were significantly associated with shorter OS time (Table 2). Radiation and temozolomide chemotherapy were significantly associated with longer OS time, indicating that PLK2 expression could be associated with MGMT methylation (Table 2). However, correlation analysis results showed that there was weak correlation between PLK2 expression and MGMT expression (*r* = 0.2696; *P*-value = 0.0007; Fig. 3A). Furthermore, PLK2 expression was not associated with MGMT methylation (*r* = 0.2373; *P*-value = 0.0936; Fig. 3B). We also found that there was weak correlation between MGMT expression and its methylation (*r* = 0.3732; *P*-value = 0.0070; Fig. 3C). Therefore, above results revealed that PLK2 expression might have a weak association with MGMT methylation in GBM. In multivariate analysis, PLK2 expression was an independent prognostic factor of poor OS (HR = 1.410, 95% CI [1.113–1.786], *P* = 0.004) in Table 2.

**Table 2:** Univariate and multivariate analyses of OS in patients with primary GBM in TCGA-GBM.

Parameters	Univariate analysis			Multivariate analysis		
	HR	95% CI	*P*	HR	95% CI	*P*
Age	1.029	[1.013–1.045]	0.000	1.015	[0.993–1.038]	0.178
Gender (female vs. male)	0.901	[0.614–1.323]	0.595	1.306	[0.775–2.201]	0.316
KPS score	0.983	[0.967–1.000]	0.053	0.994	[0.972–1.016]	0.582
Radiation (true vs. false)	0.257	[0.166–0.398]	0.000	0.540	[0.283–1.031]	0.062
Temozolomide chemotherapy (true vs. false)	0.393	[0.266–0.582]	0.000	0.449	[0.243–0.830]	0.011
PLK2 expression	1.335	[1.109–1.606]	0.002	1.410	[1.113–1.786]	0.004

**Note:**

GBM, glioblastoma multiforme; HR, hazard ratio; KPS, Karnofsky performance score.

### PLK family member expression across different subtypes and DNA copy number alterations

**Figure 4:**
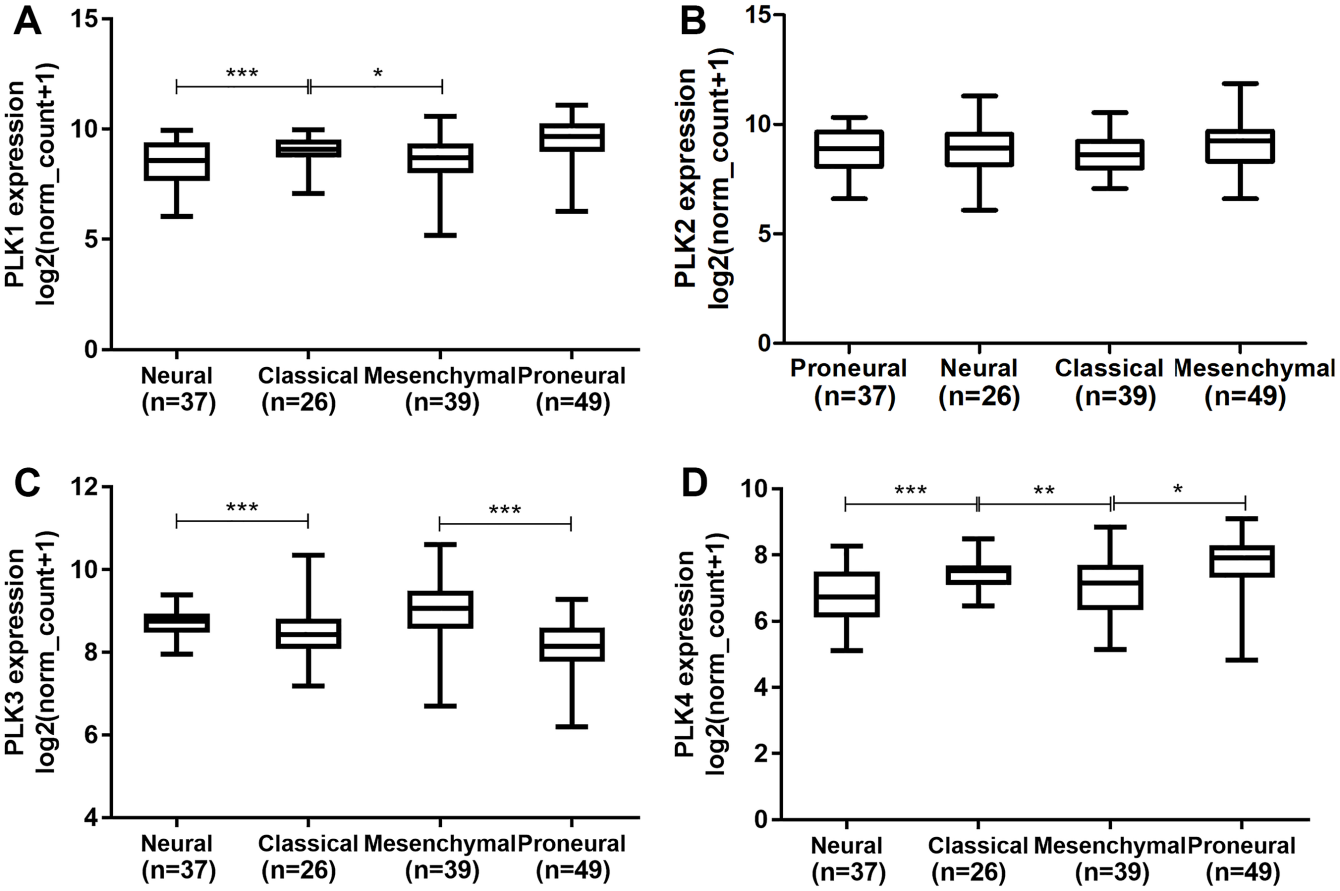
The differences of PLK family member expression across different subtypes of GBM. (A) PLK1; (B) PLK2; (C) PLK3; (D) PLK4. *P < 0.05; **P < 0.01; ***P < 0.001.

We further explored the differences of PLK2 expression across different subtypes of GBM. We first found that there was significant expression differences of PLK1 across the four subtypes (Fig. 4A). The results showed that there was no significant difference of PLK2 expression across the four subtypes including proneural (*n* = 37), neural (*n* = 26), classical (*n* = 39) and mesenchymal (*n* = 49) in Fig. 4B. Furthermore, we also observed that there were significant expression differences of PLK3 and PLK4 across the four subtypes (Figs. 4C and 4D).

**Figure 5:**
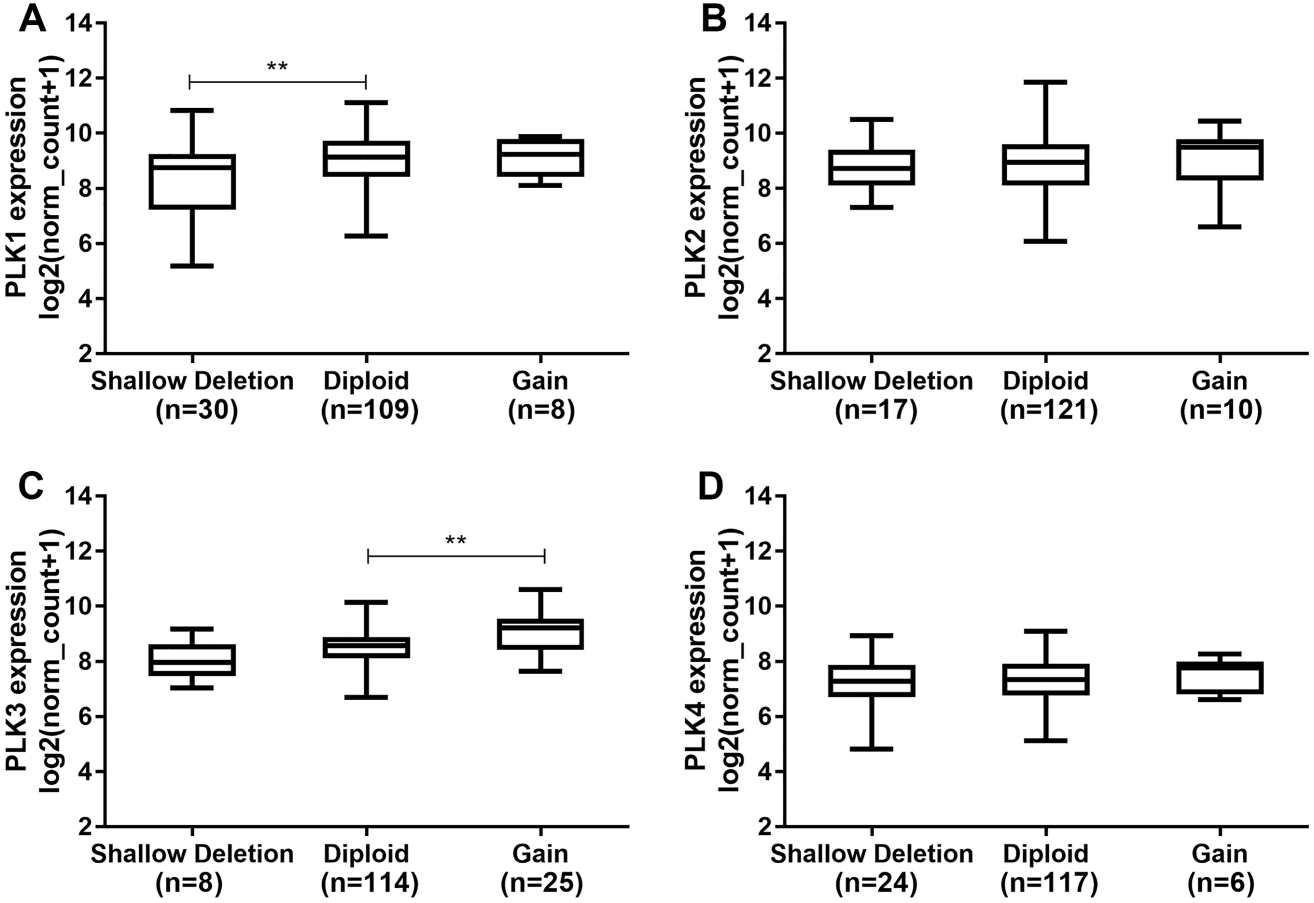
The differences of PLK family member expression across difference DNA copy number alterations. (A) PLK1; (B) PLK2; (C) PLK3; (D) PLK4. **P < 0.01.

Polo-like kinase1 expression was higher in diploid (*n* = 109) than that in shallow deletion (*n* = 30; *P* < 0.01; Fig. 5A). In addition, we found that there was no significant difference of PLK2 expression across different DNA copy number alterations including shallow deletion (*n* = 17), diploid (*n* = 121) and gain (*n* = 10; Fig. 5B). PLK3 expression was significantly higher in gain (*n* = 25) than that in diploid (*n* = 114; *P* < 0.01; Fig. 5C). We did not observed the significant differences of PLK4 expression across different DNA copy number alterations (Fig. 5D).

### PLK2 methylation might contribute to its low expression in GBM

**Figure 6:**
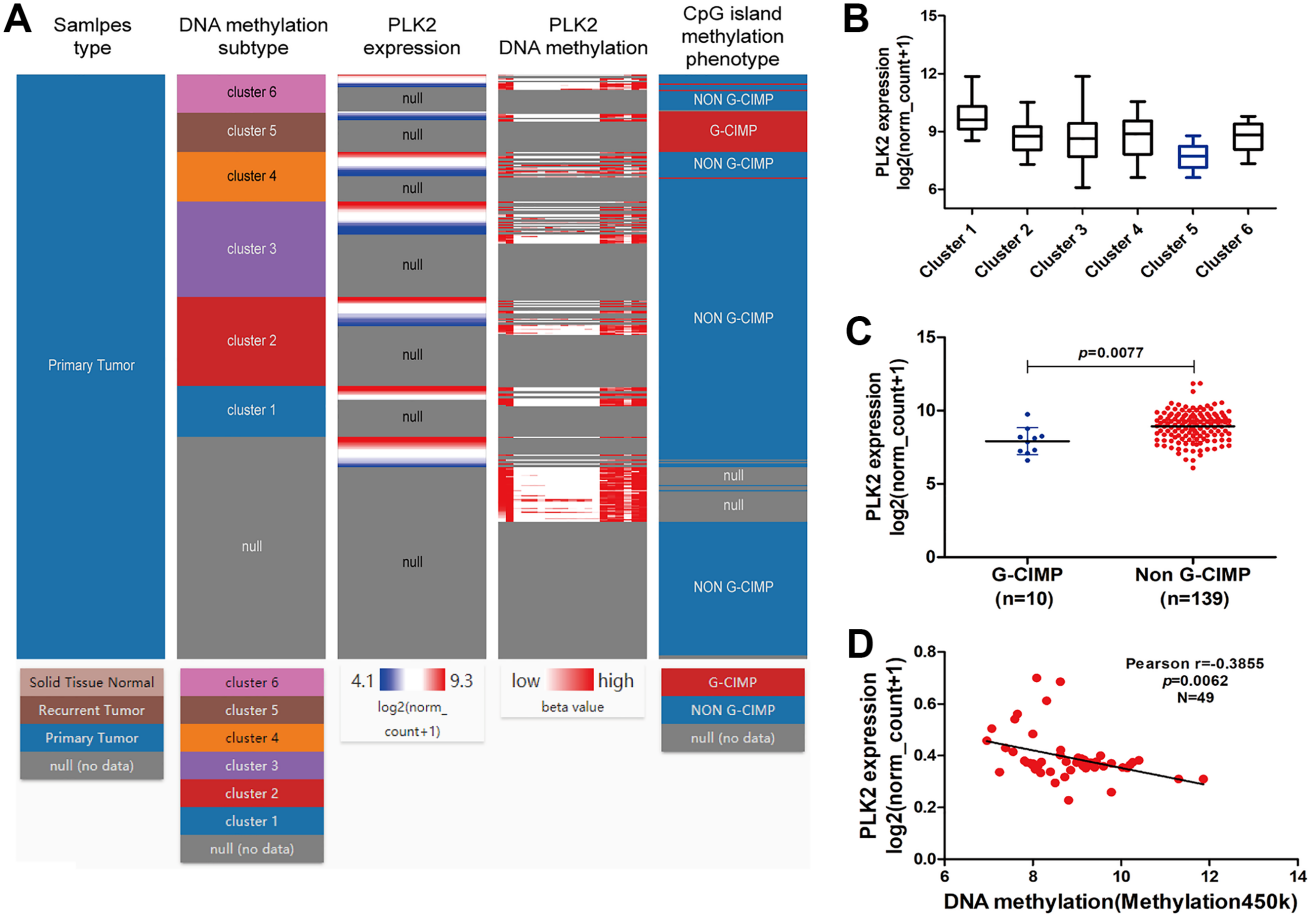
PLK2 methylation may contribute to its low expression in GBM. (A) The heat map of DNA methylation subtype (syn1701558) (cluster 1–6, the lowest to the highest), PLK2 expression, PLK2 DNA methylation (methylation 47k), CpG island methylation phenotype (including G-CIMP and non-G-CIMP); (B) The PLK2 expression in different clusters of DNA methylation; (C) The PLK2 expression in G-CIMP (*n* = 10) and non-GCIMP groups (*n* = 139); (D) Regression analysis of the correlation between PLK2 expression and its DNA methylation (*P* = 0.0062, Pearson *r* = −0.3855, *n* = 49).

Figure 6A shows the heat map of DNA methylation subtype (syn1701558) (cluster 1 to 6, the lowest to the highest), PLK2 expression (RNAseq-IlluminaHiSeq), PLK2 DNA methylation (methylation 450k), CpG island methylation phenotype (including G-CIMP and non-G-CIMP) in GBM. As depicted in Figs. 6A and 6B, in cluster 5, DNA methylated PLK2 had the lowest expression, which implied that PLK2 expression might be affected by its DNA methylation status in GBM. Moreover, DNA methylation in cluster 5 all belonged to G-CIMP. To in-depth confirm above finding, the association between PLK2 expression and G-CIMP was analyzed. The results showed that PLK2 in G-CIMP had lower expression than that in non G-CIMP group (*P* = 0.0077), in Fig. 6C. In TCGA-GBM, the correlation between PLK2 expression (*n* = 49) and its DNA methylation (methylation 45k) were measured. The regression analysis suggested that the expression of PLK2 was negatively correlated with its DNA methylation (*P* = 0.0062, Pearson *r* = −0.3855; Fig. 6D). These results revealed that PLK2 methylation may contribute to its low expression in GBM.

**Figure 7:**
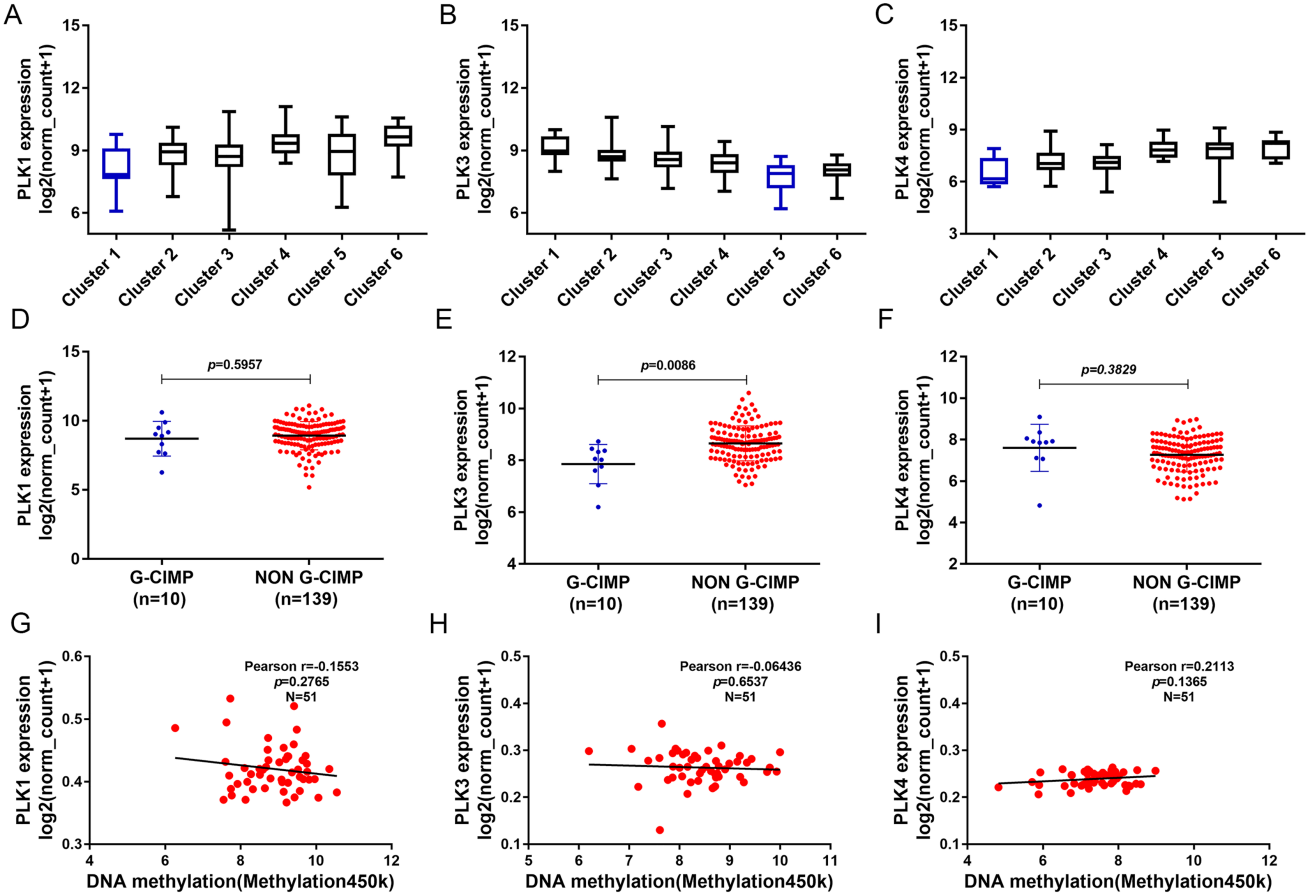
The association between PLK1, PLK3 and PLK4 expression and DNA methylation. (A–C) PLK1, PLK3 and PLK4 expression in different clusters of DNA methylation; (D–F) PLK1, PLK3 and PLK4 expression in G-CIMP (*n* = 10) and non-GCIMP groups (*n* = 139); (G–I) Regression analysis of the correlation between PLK1, PLK3 and PLK4 expression and their DNA methylation.

In addition, we found that PLK1 and PLK4 had the lowest expression in cluster 1, and PLK3 expression had the lowest expression in cluster 5 (Figs. 7A–7C). PLK3 expression was significantly higher in non G-CIMP compared with G-CIMP (*P* = 0.0086), while PLK1 and PLK4 expression was not associated with G-CIMP (Figs. 7D–7F). The regression analysis showed that PLK1, PLK3 and PLK4 all had weak correlations with DNA methylation (Figs. 7G–7I).

### Differentially expressed genes correlated with PLK2 and functional enrichment analysis

**Figure 8:**
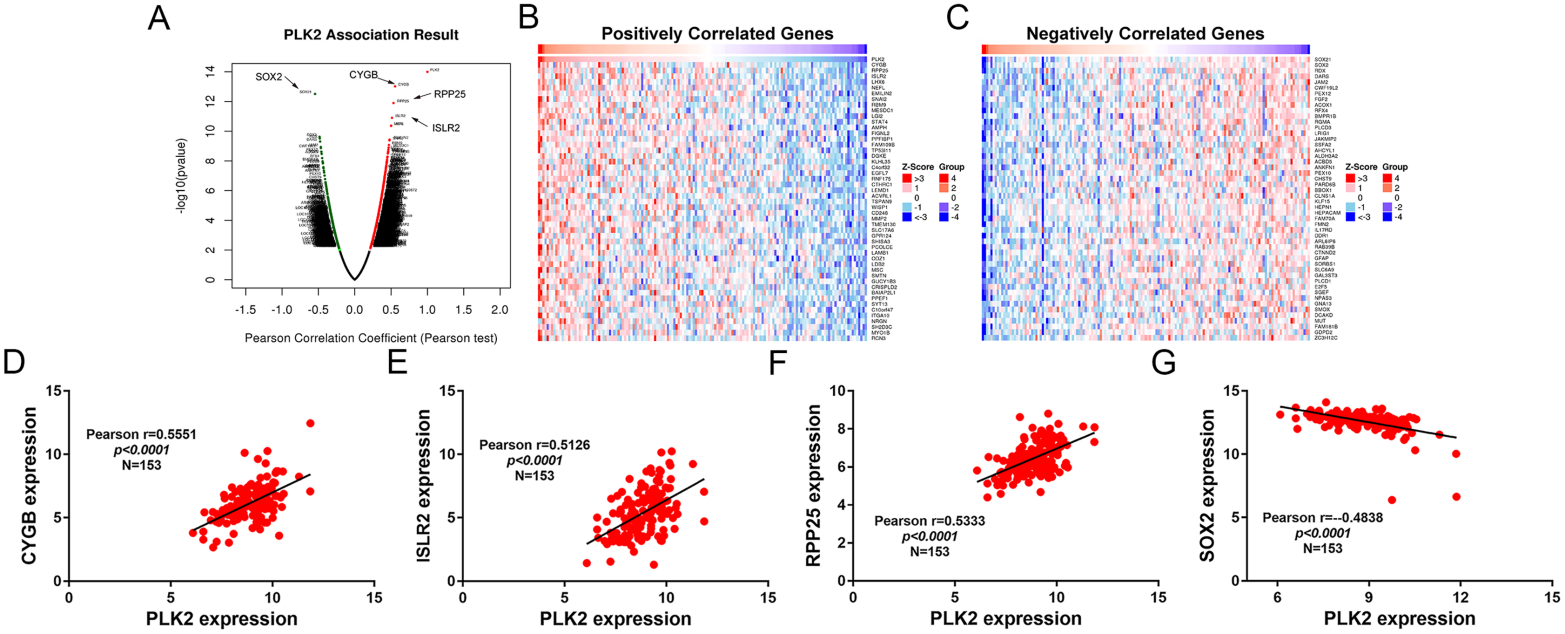
Differentially expressed genes correlated with PLK2 in GBM. (A) All differentially expressed genes correlated with PLK2; (B) The top 50 positively differentially expressed genes associated with PLK2; (C) The top 50 negatively differentially expressed genes associated with PLK2; (D–G) Scatter plots showing the significantly correlated genes including CYGB (*r* = 0.5551; *P* < 0.0001), ISLR2 (*r* = 0.5126; *P* < 0.0001), RPP25 (*r* = 0.5333; *P* < 0.0001) and SOX2 (*r* = −0.4838; *P* < 0.0001).

**Figure 9:**
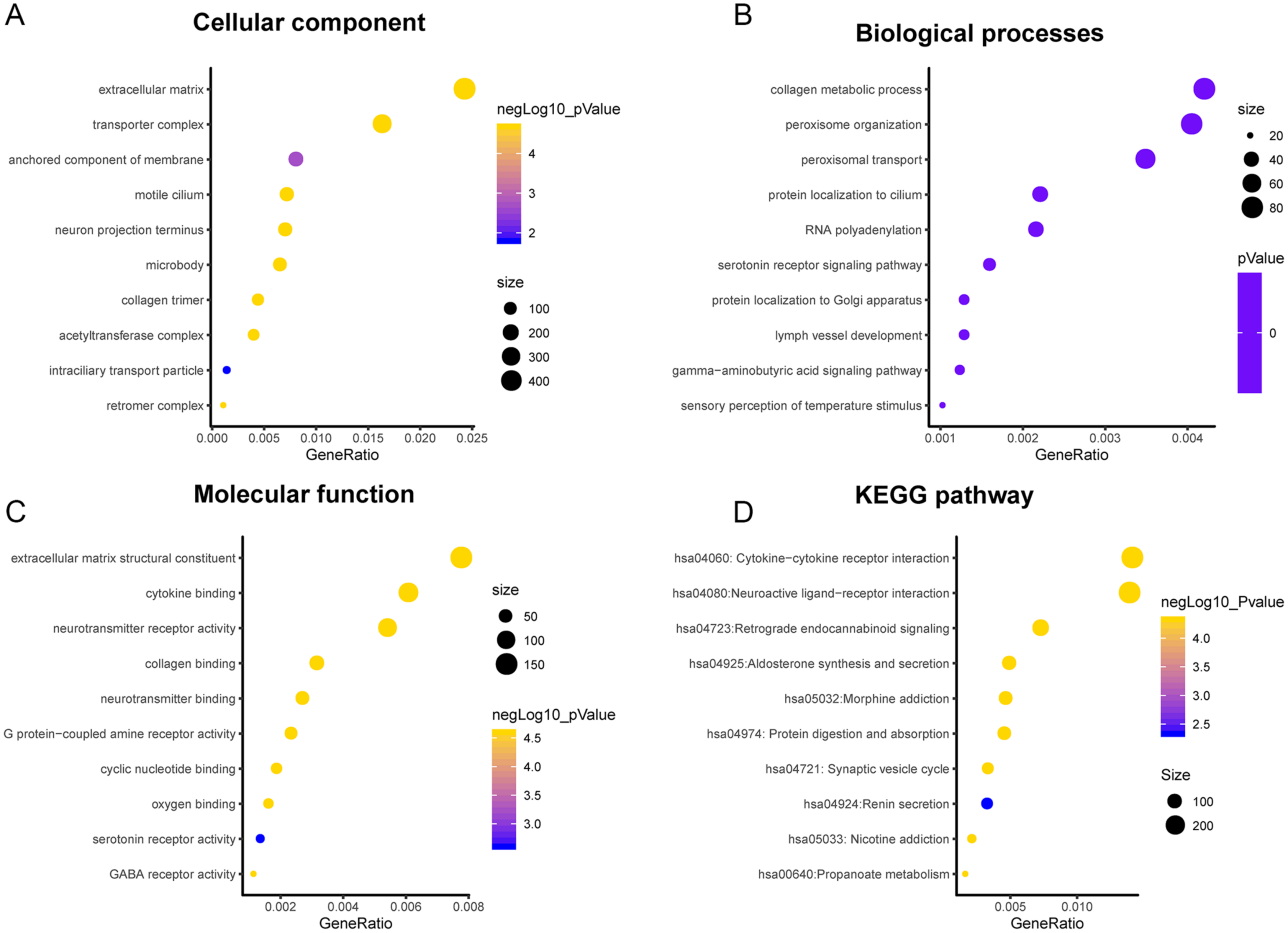
Functional enrichment analysis of differentially expressed genes correlated with PLK2. (A) Cellular component; (B) Biological processes; (C) Molecular function; (D) KEGG pathway.

A total of 3,089 differentially expressed genes (1,585 up-regulated and 1,504 down-regulated) were identified in GBM. After that, differentially expressed genes associated with PLK2 were determined, because related genes may perform similar functions in disease progression. All differentially expressed genes correlated with PLK2 are shown in Fig. 8A. The top 50 positively correlated genes and negatively correlated genes are shown in Figs. 8B and 8C respectively. Among them, CYGB (*r* = 0.5551; *P* < 0.0001), ISLR2 (*r* = 0.5126; *P* < 0.0001), RPP25 (*r* = 0.5333; *P* < 0.0001) and SOX2 (*r* = −0.4838; *P* < 0.0001) were strongly correlated with PLK2 (Figs. 8D–8G). We further observed the biological processes and pathways enriched by differentially expressed genes associated with PLK2, such as extracellular matrix (Fig. 9A), collagen metabolic process (Fig. 9B), extracellular matrix structural constituent (Fig. 9C), cytokine-cytokine receptor interaction (KEGG; Fig. 9D) and neuroactive ligand-receptor interaction (KEGG) and so on.

## Discussion

GBM is the most aggressive tumor in the central nervous system (Alexander & Cloughesy, 2017; Stupp & Newlands, 2001; Abu-Hejleh et al., 2012). Even with the advent of temozolomide, GBM patients still have a poor prognosis in part due to the presence of stem-like tumor-propagating cells that are resistant to standard therapies consisting of radiation and temozolomide (Brown et al., 2016; Lerner et al., 2015). Therefore, it is necessary to identify novel and effective therapeutic strategies for patients with GBM. In this study, our findings revealed that PLK2 expression is regulated by DNA methylation in GBM, and it could be considered to predict a favorable prognosis for patients with GBM.

Members of the PLK family have been identified as potential targets for different tumors. It has been reported that the level of PLK1 in GBM is elevated, and its inhibition limits the growth of brain cancer cells (Lee et al., 2012). Similarly, we also observed that PLK1 expression was up-regulated in GBM. Convincing evidence suggests that the clinical response to cytotoxic drugs is usually significantly higher for rapid proliferation and higher PKL2 levels of tumor cells (Mitchison, 2012; Mito et al., 2005). In our study, based on TCGA-GBM project, we found that PLK2 expression was abnormally down-regulated in patients with GBM. PLK2 expression was validated in an independent dataset. Furthermore, low PLK2 expression had a better prognosis of patients with GBM. Through univariate and multivariate analyses of OS in patients with primary GBM in TCGA-GBM, PLK2 expression could be considered as an independent prognostic indictor in patients with GBM. Additionally, we found that temozolomide chemotherapy was also associated with OS. Although temozolomide is clinically successful, its sensitivity remains a major challenge for GBM. The PLK4 inhibitor CFI400945 currently in clinical trials, has a synergistic effect with temozolomide, which increases temozolomide sensitivity in xenografts from patients with primary GBM (Zhang et al., 2019). The prognosis of four distinct molecular subtypes varies significantly (Erdem-Eraslan et al., 2013). PLK1, PLK3 and PLK4 expression had significant differences across the four subtypes. However, there was no significant difference of PLK2 expression among four subtypes including proneural, neural, classical and mesenchymal subtypes. In addition, there was no significant difference of PLK2 expression across different DNA copy number alterations including shallow deletion, diploid and gain.

It has been reported that very few patients with GBM have long term survival, which could be associated with epigenetic changes (Zhang et al., 2019). Epigenetic profiles could provide molecular biomarkers for patient prognosis. As an example, a G-CIMP positive phenotype associated with IDH1 mutations has been shown to have good prognosis for GBM (Turcan et al., 2012). Intriguingly, we found PLK2 expression was negatively correlated with its DNA methylation. CIMP, in which a large number of genes concordantly methylated, has been confirmed to be associated with OS in some types of solid tumors, such as gastric cancer, colorectal cancer, ovarian cancer, liver cancer, and glioma (Wu et al., 2010; Strathdee et al., 2001; Dahlin et al., 2010; Chen et al., 2012). Among different types of cancers, CIMP may indicate different survival outcomes. It has been reported that CIMP is enriched in the proneural subgroup and associated with favorable prognosis (Meng et al., 2015; Noushmehr et al., 2010). In our study, we found that PLK2 in G-CIMP had lower expression than that in non G-CIMP. Therefore, PLK2 expression may be modulated by its DNA methylation.

Correlated genes may perform similar functions in disease progression. In our study, we identified many differentially expressed genes correlated with PLK2. Among them, CYGB, ISLR2, RPP25 and SOX2 were strongly correlated with PLK2. CYGB is expressed in human primary GBM (Emara, Turner & Allalunis-Turner, 2014). ISLR2 and RPP25 has been confirmed as an epigenetic biomarker for GBM (Wang et al., 2018; Mock et al., 2016). Overexpression of SOX2 confers temozolomide resistance to GBM cells (Luo et al., 2019). Therefore, the four genes correlated PLK2 may participate in GBM development. Differentially expressed genes correlated with PLK2 were enriched in significant biological pathways related with the development of GBM, such as cytokine-cytokine receptor interaction and neuroactive ligand-receptor interaction and so on (Pal et al., 2018; Agrawal et al., 2018). Therefore, PLK2 could be involved in the progression of GBM.

Our study revealed that PLK2 could possess the important clinical significance for GBM prognosis. However, further molecular biological experiments are required to confirm the function of PLK2 associated with GBM. Furthermore, it is meaningful to validate the prognostic value of PLK2 methylation in a larger GBM patient cohort.

## Conclusion

In our study, we found that PLK2 expression is decreased in GBM patients, which might be regulated by its DNA methylation. Furthermore, low PLK2 expression correlates with better prognosis emphasizing its potential as a prognostic biomarker. By univariate and multivariate analyses, PLK2 could be an independent prognostic factor for GBM. Therefore, PLK2 could be considered as an independent biomarker of favorable prognosis for GBM.

## Abbreviations

GBM: glioblastoma multiforme
OS: overall survival
TCGA: The Cancer Genome Atlas
PLK: Polo-like kinase
KPS: Karnofsky Performance Score
G-CIMP: CpG island methylation phenotype
HR: hazard ratio
CIMP: CpG island methylator phenotype.

## Additional Information and Declarations

### Data Availability

The following information was supplied regarding data availability: The data of patients with GBM and the corresponding controls are available from TCGA-GBM (http://cancergenome.nih.gov/). The clinicopathological parameters of patients with primary GBM, including age, gender, Karnofsky Performance Score (KPS), Radiation therapy, temozolomide chemotherapy and living status are available from the UCSC Xena Browser (http://xena.ucsc.edu/).
